# Subacute‐onset cataract in a 29‐year‐old man with mitochondrial encephalomyopathy: A case report

**DOI:** 10.1002/ccr3.8417

**Published:** 2024-01-04

**Authors:** Lu Chen, Yueyang Zhong, Jingjie Xu, Qiuli Fu, Ke Yao

**Affiliations:** ^1^ Eye Center of the Second Affiliated Hospital, School of Medicine Zhejiang University Hangzhou Zhejiang China; ^2^ Zhejiang Provincial Key Lab of Ophthalmology Hangzhou Zhejiang China

**Keywords:** case report, cataract, mitochondrial encephalomyopathy, ocular manifestation

## Abstract

This case report aims to emphasize that subacute occurrence of nuclear cataract might be one of the underestimated manifestations of mitochondrial encephalomyopathy, thus periodical ophthalmologic examinations are recommended.

## Graphical abstract

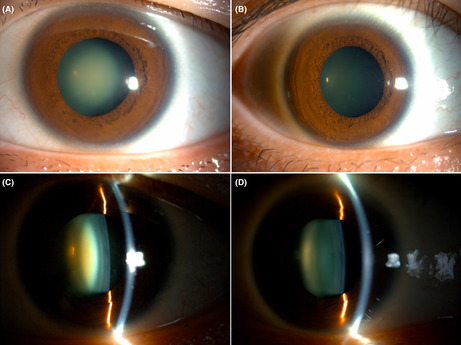

## 1 INTRODUCTION

Mitochondrial disease, a heterogeneous group of disorders related to energy metabolism, is caused by mutation of the mitochondrial or nuclear DNA.^1^ As one of these multisystem disorders, mitochondrial encephalomyopathy (ME) combines mitochondrial myopathy with a complex array of neurodegenerative disease symptoms, primarily affecting the nervous system and muscles, whose manifestations regarding lens are rarely mentioned.^1^ Here, we describe an uncommon case of subacute‐onset nuclear cataract in adult ME patient with detailed ocular presentations for the first time. We present the following case in accordance with the CARE reporting checklist.

## 2 CASE REPORT

**FIGURE 1:**
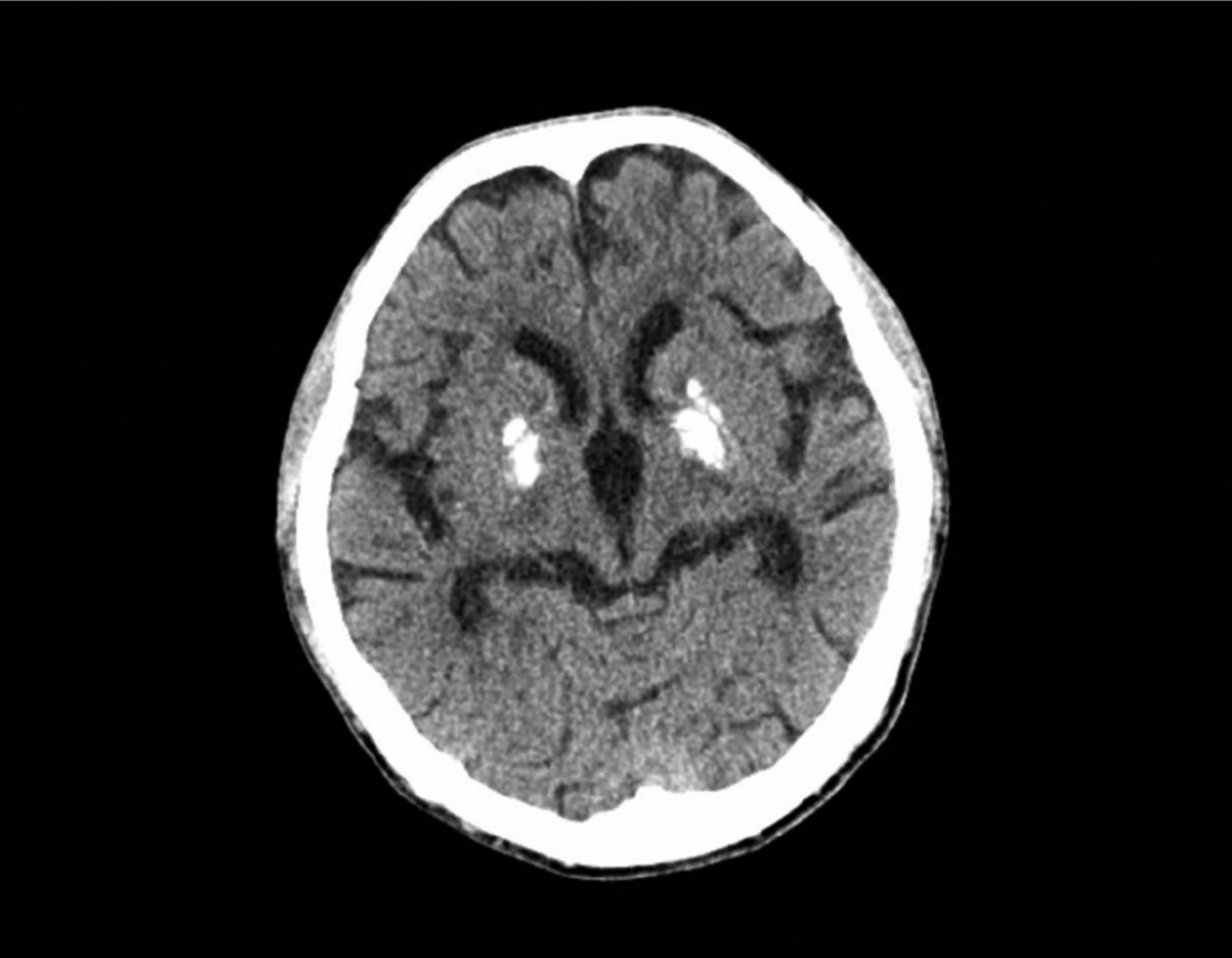
Head computed tomography (CT) scan revealing calcification in bilateral basal ganglia.

A 29‐year‐old man without previous symptoms initially presented with blurred vision for the past 4 months. He was diagnosed with ME at the age of 24 (with dizziness as chief complaint, no stroke‐like episodes, muscle biopsy showing broken red fibers, and refusal of genetic testing), and prescribed with vitamin B_2_, vitamin E, mecobalamin, idebenone, lorazepam, and levetiracetam tablets for long‐term use. His head computed tomography (CT) showed calcification in bilateral basal ganglia (Figure 1). He denied previous ocular surgeries, trauma, or family history of congenital cataract.

**FIGURE 2:**
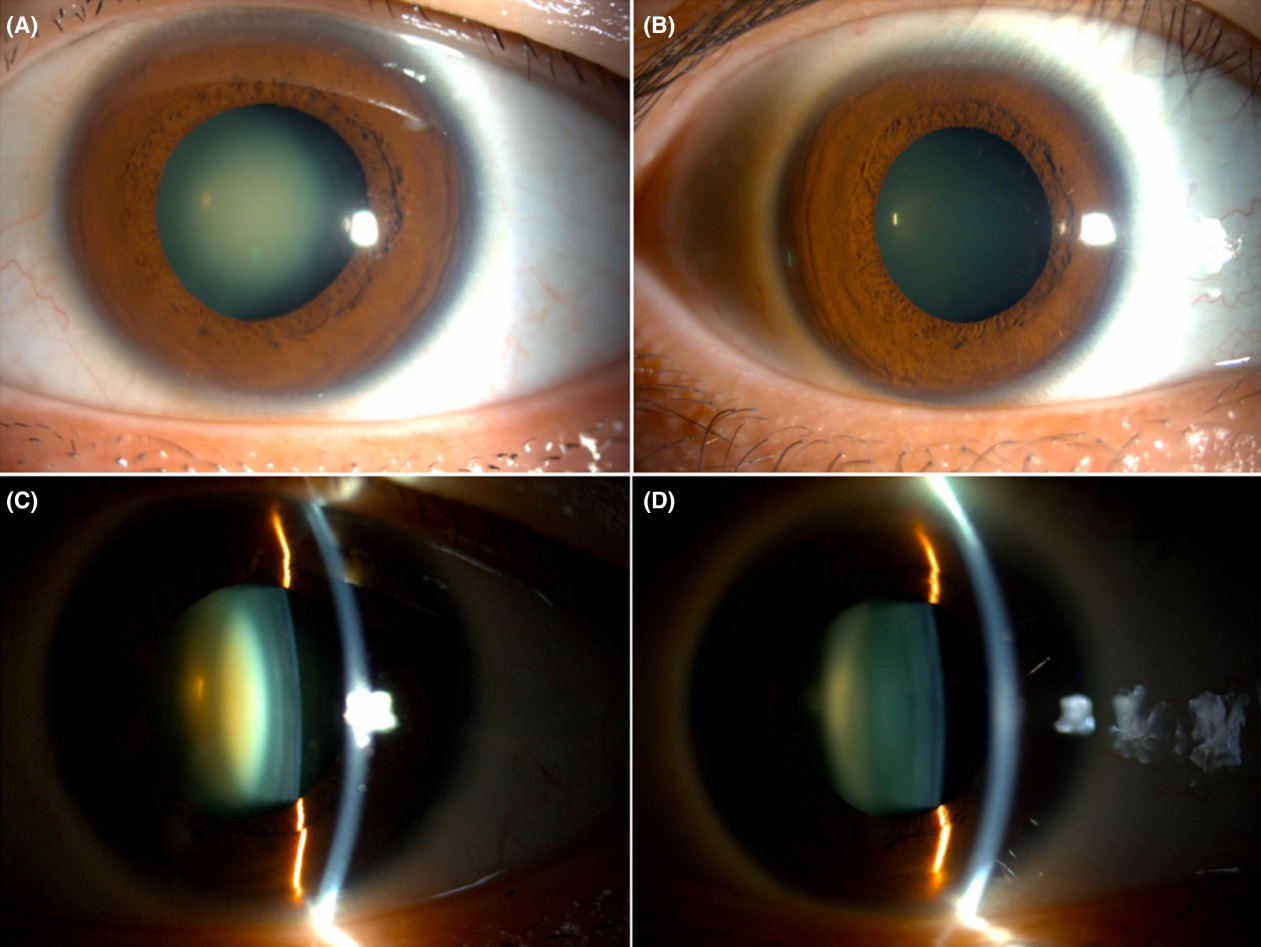
Slit lamp photograph of the left eye (LE) (A, C) and the right eye (RE) (B, D) demonstrating dense 3+ nuclear cataract in the LE and 1+ nuclear cataract in the RE.

**FIGURE 3:**
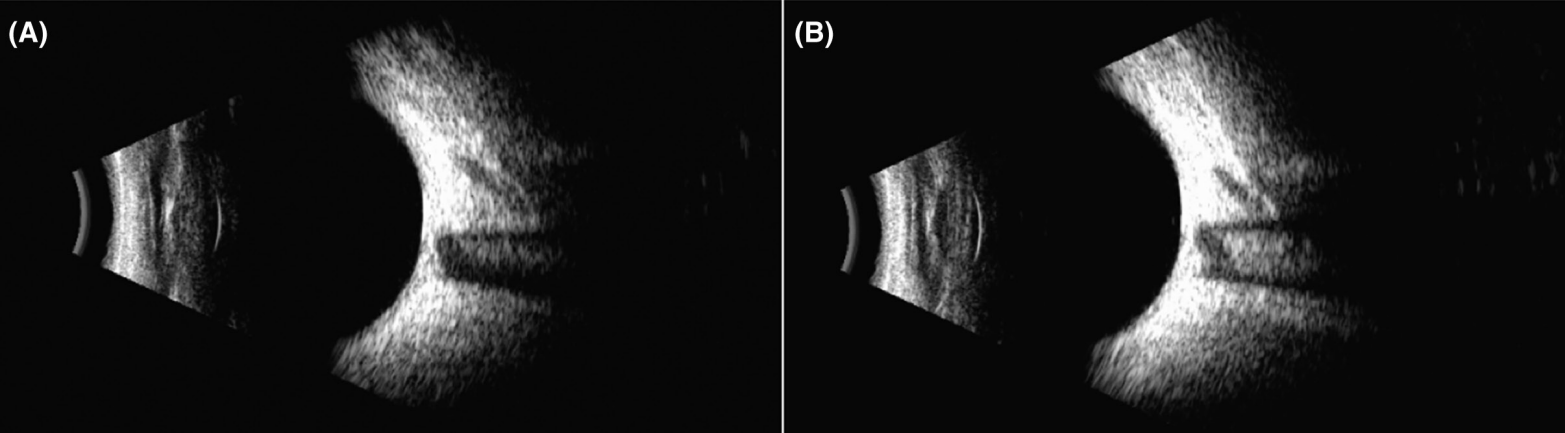
B‐scan ultrasonography of the left eye (LE) (A) and the right eye (RE) (B) revealed no characteristic changes.

His best‐corrected visual acuity (VA) was 20/32 in the right eye (RE) and 20/40 in the LE. The intraocular pressures were 14.5 and 13.0 mmHg, respectively. Slit lamp photograph revealed 1+ nuclear cataract in the RE lens and significant 3+ nuclear cataract in the LE lens (Figure 2). B‐scan ultrasonography revealed no characteristic changes (Figure 3). Ocular movements were considered normal. No corneal or fundal abnormalities were noted. Upon diagnosis of cataract, his preadmission blood tests for cataract surgery suggested high levels of lactate (2.41 mmol/L, controls 0.7–2.1 mmol/L) and creatine kinase (338 U/L, controls < 164 mmol/L).

**FIGURE 4:**
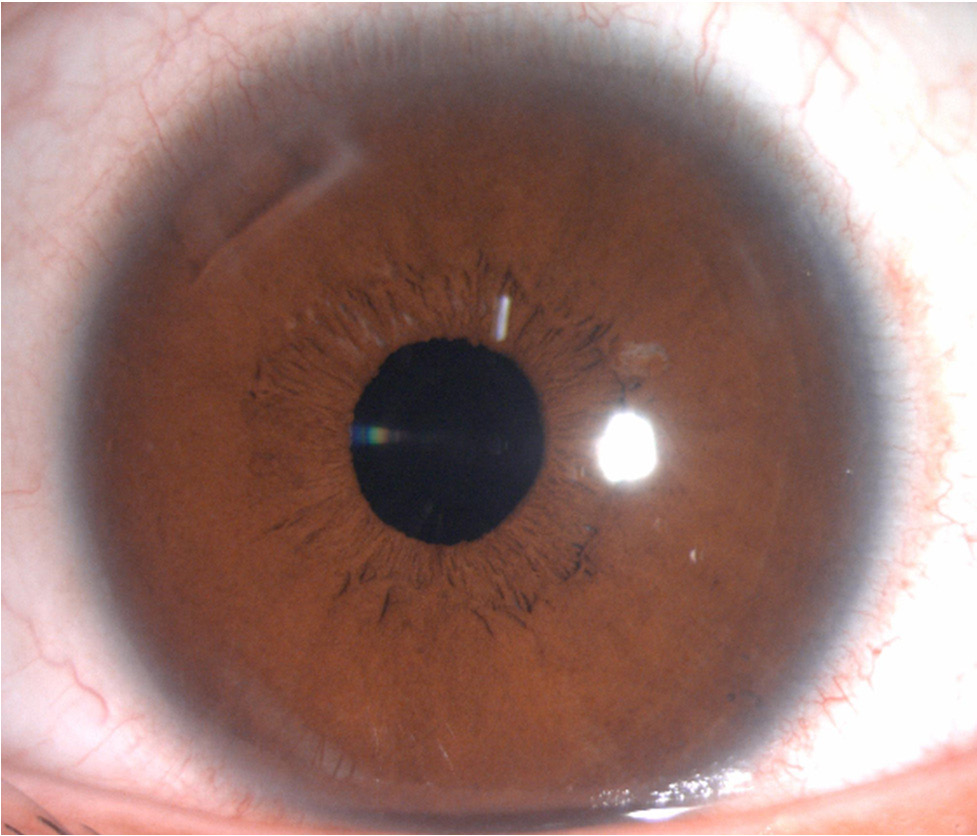
Slit lamp photograph of the left eye (LE) at 1 month after the cataract surgery.

**FIGURE 5:**
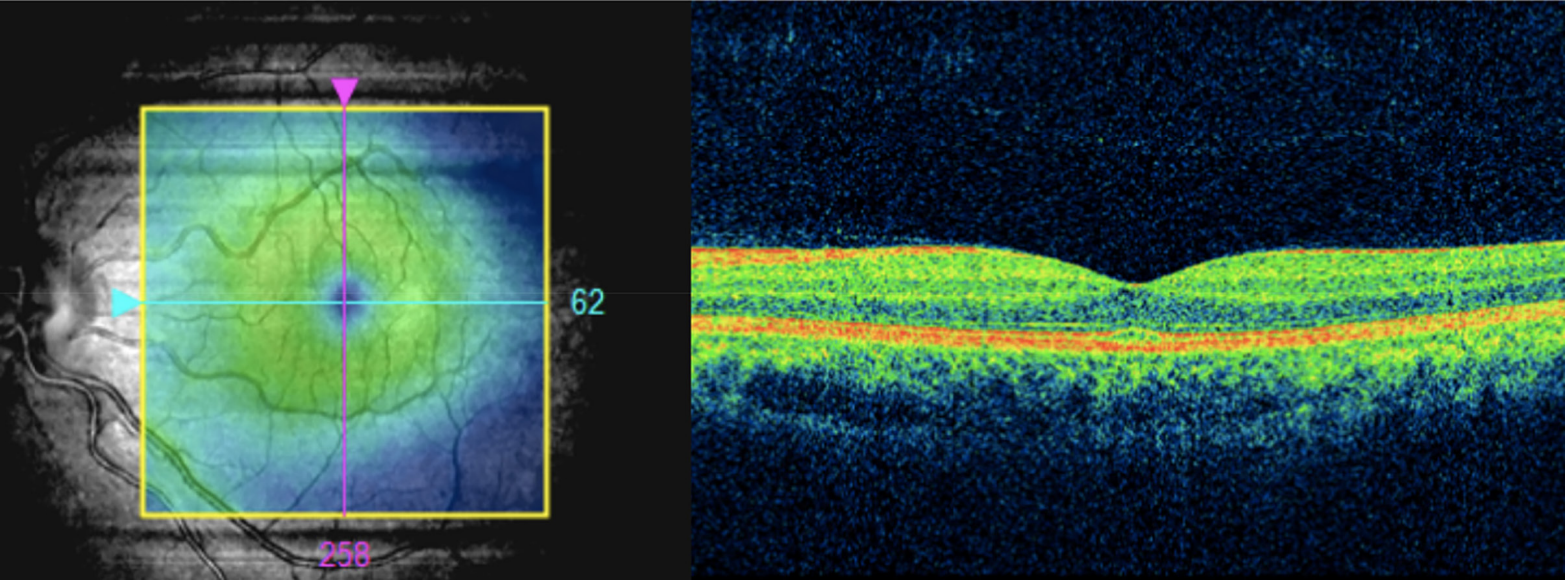
Optical coherence tomography of the left eye (LE) at 1 month after the cataract surgery.

Femtosecond laser‐assisted cataract surgery with implantation of a monofocal intraocular lens (IOL) was performed in the LE. The cataract surgery went smoothly. Seven days postoperatively, the visual acuity (VA) in the LE improved to 20/20. At the 1 month follow‐up, VA was stable at 20/20 in the LE. Postoperative anterior segment of the LE was normal (Figure 4). And its macular optical coherence tomography was within normal limits (Figure 5).

## 3 DISCUSSION

Presented here is a case of a 29‐year‐old man, who developed subacute‐onset nuclear cataract with 5‐year history of ME. Nuclear cataracts were noted in both eyes, but were far more significant in the center of the LE, which progressed to the point requiring cataract surgery to restore vision.

Tissues with high energetic demands such as extraocular muscles and retina are more likely to be affected in mitochondrial diseases, thus the most common ocular manifestations of mitochondrial disease are optic atrophy, pigmentary retinopathy, and ophthalmoplegia.^2^ Cataract is not referred as one of the common complications of ME, only one case mentioned cataract in an 18‐year‐old ME patient without detailed ocular information.^3^

In terms of this case, subacute occurrence of nuclear cataract might be one of the underestimated ocular manifestations of ME. Mitochondrial damage in ME patients increases the production of reactive oxygen species (ROS). An excessive amount of ROS damages the lens proteins, resulting in protein aggregation, which affects the transparency of the lens.^4^ Considerable evidence showed that, mitochondrial dysfunction and ROS imbalance are important causes of age‐related cataract.^5^ Besides, ME is associated with extreme genetic heterogeneity, and different mutations correspond to different clinical manifestations. It is possible that the unknown gene mutation in this case may also contribute to the early‐onset cataract.

Another less possible explanation is drug‐induced cataract. Nevertheless, no evidence has been found to related vitamin B_2_, vitamin E, mecobalamin, idebenone, lorazepam, or levetiracetam with higher risk of cataract. Besides, nuclear cataracts in this case were dissimilar to the typical drug‐induced cataracts with mainly equatorial and posterior subcapsular changes.^6^

## 4 CONCLUSION

This report highlights subacute cataract formation in adult ME patient, which may be one of the underestimated ocular manifestations of this mitochondrial disease. Long‐term use of corresponding medication might also be attributable. We recommend that clinicians be aware of potential ocular abnormalities in patients alike and arrange periodical examinations.

## AUTHOR CONTRIBUTIONS

**Lu Chen:** Investigation; writing – original draft. **Yueyang Zhong:** Writing – review and editing. **Jingjie Xu:** Data curation; investigation. **Qiuli Fu:** Investigation. **Ke Yao:** Conceptualization; project administration.

## FUNDING INFORMATION

This work is supported by National Natural Science Foundation of China (81670833, 81870641, 8207939, 81300641, 82271063, 82201158), Zhejiang Province Key Research and Development Program (2019C03091, 2020C03035), and Fundamental Research Funds of the Central Universities (2019QNA7026).

## CONFLICT OF INTEREST STATEMENT

The authors declare that they have no conflict of interest.

## CONSENT

Written informed consent was obtained from the patient to publish this report in accordance with the journal's patient consent policy.

## Data Availability

There are no data generated from this study.
